# Clinical characteristics, risk factors, and prognostic modeling for poor outcomes in children with influenza-associated encephalopathy: A retrospective cohort study

**DOI:** 10.1097/MD.0000000000046932

**Published:** 2026-01-16

**Authors:** Fei Xiao, Xingfeng Cheng, Yuanmei Shi, Hong Cheng, Hong Zhang, Hui Li, Kang Xu

**Affiliations:** a Department of Pediatrics, Taixing People’s Hospital Affiliated to Yangzhou University, Taizhou, China; b Department of Pediatric Intensive Care Unit, Wuhan Children’s Hospital (Wuhan Maternal and Child Healthcare Hospital), Tongji Medical College, Huazhong University of Science and Technology, Wuhan, China; c Key Laboratory of Experimental and Translational Non-coding RNA Research, Yangzhou University, Yangzhou, China; d Department of Epidemiology and Statistics, Taixing People’s Hospital Affiliated to Yangzhou University, Taizhou, China.

**Keywords:** influenza-associated encephalopathy, neurological sequela, pediatric intensive care, prognostic factors, risk prediction model

## Abstract

Influenza-associated encephalopathy (IAE) in children is a rare but severe complication associated with high morbidity and mortality. Timely identification of high-risk cases remains challenging due to variable clinical presentations and limited pediatric-specific evidence. This retrospective cohort study included 198 children diagnosed with IAE between 2015 and 2022 in a pediatric intensive care unit. Patients were categorized by in-hospital prognosis, defined as either poor (including death, coma, or neurological sequelae at discharge) or favorable outcome. Clinical and laboratory parameters were compared between groups. Logistic regression identified independent risk factors, which were used to construct a prognostic model and corresponding nomogram. Discriminatory performance was evaluated using receiver operating characteristic analysis. Among the 198 patients (median age 26 months), 88 (44.4%) met criteria for poor prognosis. These included death (21.6%) and sequelae such as cognitive impairment (23.9%), motor dysfunction (20.5%), and epilepsy (15.9%). Poor outcome was significantly associated with lower Glasgow Coma Scale scores (median 7 vs 12, *P* < .001), cranial magnetic resonance imaging abnormalities (71.6% vs 39.1%, *P* < .001), acute respiratory distress syndrome, hyperglycemia, and elevated brain natriuretic peptide levels. Multivariable logistic regression identified 5 independent predictors: Glasgow Coma Scale ≤ 8 (adjusted odds ratio [aOR] 3.42, 95% confidence interval [CI]: 1.76–6.64), abnormal magnetic resonance imaging (aOR 3.25, 95% CI: 1.59–6.67), acute respiratory distress syndrome (aOR 2.92, 95% CI: 1.46–5.85), glucose > 8.3 mmol/L (aOR 2.51, 95% CI: 1.23–5.12), and brain natriuretic peptide > 100 pg/mL (aOR 1.98, 95% CI: 0.96–4.12). The model showed strong predictive performance (area under the curve = 0.84, sensitivity of 82.3%, and specificity of 88.2% at the optimal cutoff value). Among children with IAE, nearly half experienced poor in-hospital outcomes. A combination of neurological severity, radiologic findings, respiratory status, and metabolic derangements independently predicted prognosis. These findings may aid early risk stratification and clinical decision-making in pediatric critical care.

## 1. Introduction

Influenza-associated encephalopathy (IAE) represents a rare but devastating complication of seasonal influenza, primarily affecting children.^[1,2]^ Although its incidence is low, IAE accounts for a disproportionate share of influenza-related mortality and long-term neurological disability in pediatric populations.^[3]^ The onset is often abrupt, with rapidly progressing neurological dysfunction such as altered consciousness or seizures, frequently in the absence of overt central nervous system infection.^[4]^ The pathophysiology is thought to involve a para-infectious inflammatory cascade and systemic immune dysregulation, rather than direct viral invasion, contributing to diagnostic complexity and clinical unpredictability.^[5]^

Despite improvements in influenza surveillance and critical care capacity, early identification of children at high risk for adverse outcomes remains a substantial challenge.^[6,7]^ Existing literature on pediatric IAE is largely confined to small case series or descriptive analyses, often lacking adequate prognostic stratification.^[1,3]^ Mortality is frequently reported, but functional sequelae at discharge – such as cognitive delay, epilepsy, or motor deficits – are rarely quantified, and few studies have attempted to incorporate these into unified outcome definitions. Furthermore, most available data originate from high-income countries,^[8,9]^ with limited applicability to healthcare contexts such as China, where resource constraints and disease presentation patterns may differ.

Recent advances in virological diagnostics and intensive care protocols have improved the survival of critically ill children with viral encephalopathies.^[10]^ Nevertheless, clinical management of IAE remains largely reactive rather than anticipatory. The pathogenesis of IAE is believed to be predominantly immunologically mediated, involving cytokine dysregulation and blood–brain barrier disruption, rather than direct neurotropic viral invasion. This often results in abrupt and fulminant neurological deterioration in previously healthy children, with minimal prodromal warning.^[11]^ Because clinical, biochemical, and radiologic presentations are heterogeneous and nonspecific in the early phase, frontline clinicians frequently lack reliable criteria to distinguish children at risk of poor neurological recovery from those likely to experience full resolution.^[12,13]^ In such a context, outcome prediction depends heavily on experience and intuition rather than evidence-informed frameworks.

Moreover, existing efforts to predict outcomes in pediatric encephalopathy have either focused on etiology-specific cohorts – such as acute necrotizing encephalopathy or viral encephalitis – or employed broad neurocritical care populations that dilute syndrome-specific characteristics.^[14]^ Within IAE, prior research has predominantly centered on severe subtypes or fatal cases, often neglecting children who survive with subtle or delayed impairments. Crucially, there remains no widely accepted, operationalized definition of “poor prognosis” in IAE that captures both fatal and disabling outcomes. The absence of such a composite endpoint impairs the comparability and clinical utility of existing data. In resource-constrained environments, where neuroimaging or specialist consultations may not be readily available, there is an urgent need for pragmatic, data-driven tools that enable early prognostic stratification based on information accessible at the bedside.

In this study, we aimed to characterize the clinical features and outcomes of children diagnosed with IAE over an 8-year period in a high-volume pediatric intensive care unit (PICU). Using a rigorously defined composite endpoint encompassing mortality, persistent coma, and neurological sequelae, we sought to identify independent prognostic factors and evaluate their predictive utility through a multivariable model. By integrating routinely available clinical indicators, this study intends to support timely risk stratification and inform early intervention strategies for children with this severe neurological complication of influenza.

## 2. Materials and methods

### 2.1. Study design and setting

This investigation was structured as a retrospective observational cohort study conducted at the PICU of a tertiary teaching hospital in China, designated as a national referral center for severe pediatric infectious and neurological diseases. All eligible cases were drawn from a consecutive series of admissions between January 2015 and December 2022, a period selected to ensure both adequate sample size and consistency in diagnostic and treatment protocols. The study adhered to the methodological and reporting standards recommended by the Strengthening the Reporting of Observational Studies in Epidemiology (STROBE) statement.

The electronic medical record system of the hospital, fully integrated with the laboratory, radiological, and pharmacy databases, was used to extract clinical and diagnostic data. Case identification and confirmation were independently performed by 2 pediatric intensivists, with disagreements resolved by a senior pediatric neurologist to minimize classification bias. The study was approved by Wuhan Children’s Hospital Medical Ethics Committee (approval number: 2024R044-E01), and the requirement for informed consent was formally waived due to the retrospective nature of the data and anonymized processing framework. All study procedures adhered to the principles outlined in the Declaration of Helsinki.

### 2.2. Patient selection and case definition

Children were eligible for inclusion if they were admitted to the PICU during the study period with a diagnosis of IAE. Inclusion criteria encompassed: age between 1 month and 14 years at admission; laboratory-confirmed influenza virus infection via nasopharyngeal swab reverse-transcription polymerase chain reaction (RT-PCR), regardless of influenza virus serotype (type A or B), and irrespective of current season or previous influenza vaccination history; and acute onset of central nervous system dysfunction manifesting as altered consciousness, seizures, or behavioral disturbances not attributable to alternative etiologies. Exclusion criteria included: time from influenza symptom onset to PICU admission exceeding 7 days; laboratory-confirmed coinfection with other major pathogens besides the influenza virus (such as respiratory syncytial virus, adenovirus, parainfluenza virus, herpes simplex virus, etc); presence of definite underlying non-influenza-related encephalopathies, such as severe traumatic brain injury, known sequelae of viral encephalitis, congenital metabolic diseases, or neurodevelopmental disorders; or insufficient documentation regarding clinical outcomes at discharge.

The operational definition of IAE was consistent with the diagnostic framework proposed by the World Health Organization and refined through national consensus guidelines. Diagnosis required the coexistence of virologically confirmed influenza infection and acute encephalopathic manifestations, supported where available by neuroimaging, electroencephalography, or cerebrospinal fluid (CSF) findings excluding other infectious causes. To ensure diagnostic specificity, all cases were independently verified by 2 pediatric infectious disease specialists and 1 pediatric neurologist, blinded to outcome status.

Patients were stratified post hoc into 2 groups based on discharge outcomes: those who experienced poor prognosis and those with favorable recovery. Poor prognosis was operationally defined as the occurrence of any of the following: in-hospital mortality, persistent coma or vegetative state, or new-onset neurological sequelae at discharge, including but not limited to limb paralysis, speech deficits, or refractory epilepsy. Given that multiple adverse events could occur in the same patient, group assignment was based on the presence of any qualifying poor outcome indicator.

### 2.3. Data collection and variables assessed

Clinical and laboratory data were retrospectively retrieved from the hospital’s integrated electronic medical record system by trained data abstractors using a standardized data extraction template. Variables were collected at the time of PICU admission or within the first 24 hours of hospitalization to capture baseline severity and reduce temporal confounding. Where applicable, abnormal values were confirmed across multiple time points to exclude transient fluctuations attributable to interventions or measurement error.

Demographic variables included age, sex, weight, and seasonal distribution of onset. Baseline physiological parameters encompassed systolic and diastolic blood pressure (mm Hg), heart rate (beats/min), body temperature (°C), and peripheral oxygen saturation (SpO₂, %). Laboratory indices captured included complete blood count, blood glucose (mmol/L), serum creatinine (µmol/L), alanine aminotransferase (U/L), brain natriuretic peptide (BNP, pg/mL), and C-reactive protein (mg/L). Neurological severity was assessed using the Glasgow Coma Scale (GCS), dichotomized at ≤8 to reflect clinically relevant thresholds for coma. Imaging data (cranial magnetic resonance imaging [MRI]) of all pediatric patients were independently reviewed by 2 radiologists who were blinded to the clinical outcomes. “Abnormal imaging findings” specifically refer to observations associated with acute encephalopathy, including but not limited to: acute thalamic necrosis (often bilateral and symmetrical), cerebral edema, signal abnormalities in the cerebral cortex or white matter, restricted diffusion foci on diffusion-weighted imaging, and involvement of the brainstem or basal ganglia. For all pediatric patients who underwent lumbar puncture, CSF samples were tested for influenza virus using RT-PCR to exclude direct invasion of the central nervous system by the influenza virus (i.e., influenza viral encephalitis). The IAE defined in this study primarily refers to noninvasive acute encephalopathy. Therefore, only cases with negative CSF influenza virus RT-PCR results were included in the final analysis.

Preexisting comorbidities were also recorded, including congenital heart disease, epilepsy, and chronic lung disease. Organ dysfunctions during PICU stay, including the development of acute respiratory distress syndrome (ARDS), shock, and renal impairment, were documented using standard pediatric critical care definitions. Therapeutic interventions such as antiviral administration, intravenous immunoglobulin (IVIG), corticosteroid therapy, and mechanical ventilation were extracted and coded based on administration within 48 hours of admission. All variable definitions and coding schemes were pre-specified prior to data analysis to reduce outcome-dependent misclassification.

### 2.4. Outcome measures and prognostic endpoint

The primary outcome of interest was defined as a composite measure of poor prognosis at the time of hospital discharge. This composite endpoint comprised 3 mutually non-exclusive adverse clinical events: in-hospital mortality; persistent vegetative state or unresponsive coma; and new-onset neurological sequelae as assessed by treating physicians and verified through discharge documentation. Neurological sequelae were subcategorized into motor dysfunction (e.g., hemiplegia, spasticity), cognitive impairment (e.g., developmental regression, language delay), and refractory seizures requiring maintenance antiepileptic therapy.

Neurological outcomes at discharge were assessed using the pediatric cerebral performance category scale. This scale is a widely utilized standardized tool in the field of pediatric critical care, with the following categories: score 1 (normal), score 2 (mild disability), score 3 (moderate disability), score 4 (severe disability), score 5 (coma or vegetative state), and score 6 (death). For dichotomous outcome analysis, a pediatric cerebral performance category scale score of 3 to 6 was defined as “poor prognosis,” while a score of 1 to 2 was defined as “favorable outcome.” This stratification reflected clinically meaningful distinctions in recovery trajectories and resource utilization following PICU discharge. Given the retrospective nature of the study and absence of long-term follow-up, the endpoint was constrained to discharge status, thereby ensuring data completeness while preserving prognostic validity.

Secondary measures included the distribution and frequency of individual poor outcome components, allowing disaggregated interpretation of the event burden. Additionally, differences in therapeutic approaches between outcome groups were evaluated, particularly the use of antiviral agents, immunomodulatory treatments, and supportive interventions such as invasive ventilation. These secondary endpoints provided ancillary insight into potential treatment-effect heterogeneity within the critically ill IAE population.

### 2.5. Statistical analysis

All statistical analyses were performed using R software (version 4.2.2) and SPSS (version 26.0, IBM Corp., Armonk). Continuous variables were tested for normality using the Shapiro–Wilk method. Normally distributed variables were summarized as mean ± standard deviation and compared between groups using the independent samples *t*-test; non-normally distributed variables were reported as median with interquartile range and compared using the Mann–Whitney *U* test. Categorical variables were expressed as counts with proportions and analyzed using the chi-square test or Fisher’s exact test, as appropriate.

Univariate logistic regression analysis was first applied to screen potential prognostic factors for poor outcomes. Variables with a *P*-value < .1 in univariate models or with established clinical relevance were subsequently entered into a multivariable logistic regression model. Multicollinearity among predictors was assessed using variance inflation factors, with a threshold of < 5 set for inclusion. Final variable selection was performed using backward stepwise elimination based on the Akaike information criterion. Adjusted odds ratios (aORs), 95% confidence intervals (CIs), and corresponding *P*-values were reported for each retained covariate.

Model discrimination was evaluated by receiver operating characteristic (ROC) curve analysis, and the area under the curve was used as the primary measure of diagnostic performance. In addition, the optimal cutoff value was determined using the Youden index, with corresponding sensitivity and specificity values being reported. A visual nomogram was constructed based on the final logistic model coefficients using the rms package in R. The clinical utility of the nomogram was further assessed by calibration plots and bootstrap internal validation (1000 resamples) to evaluate the agreement between predicted and observed probabilities. A 2-tailed *P*-value < .05 was considered statistically significant in all analyses unless otherwise specified.

## 3. Results

### 3.1. Baseline characteristics and clinical profiles of the cohort

**Figure 1.**
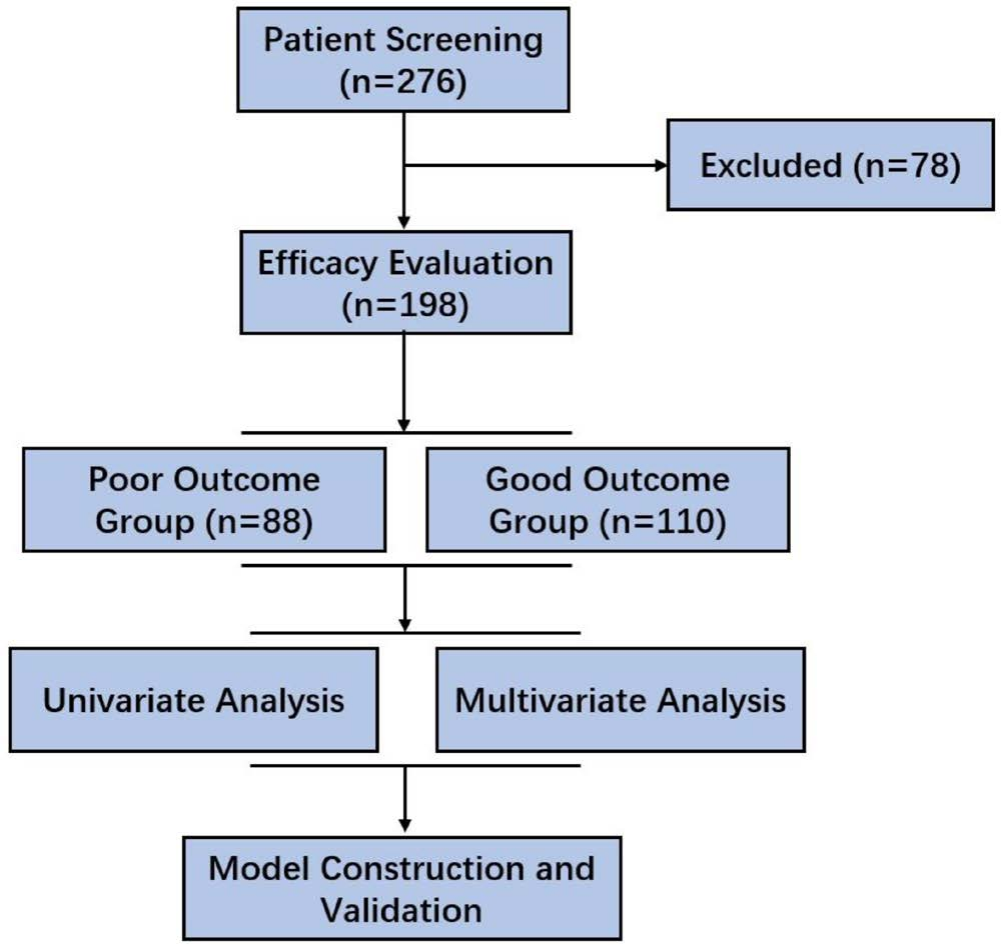
Study flow diagram. Flowchart illustrating patient selection and inclusion process for the retrospective cohort. A total of 237 pediatric patients diagnosed with IAE were initially screened from the PICU database between January 2015 and December 2022. After excluding cases with incomplete records, non-IAE diagnoses, or age outside the inclusion criteria, 198 children were included in the final analysis. Patients were classified into poor outcome and favorable outcome groups based on a composite endpoint comprising death, persistent coma, or neurological sequelae at discharge. IAE = influenza-associated encephalopathy, PICU = pediatric intensive care unit.

Among 276 pediatric patients admitted to the intensive care unit with suspected influenza-associated neurological complications during the surveillance period, 198 met diagnostic and clinical inclusion criteria for IAE and were retrospectively enrolled. Patients were categorized into 2 prognostic subgroups based on in-hospital outcomes: those with unfavorable prognosis, defined by death or the presence of neurological sequelae at discharge, and those with favorable recovery. The poor outcome group comprised 88 children, while the remaining 110 were assigned to the favorable outcome group (Fig. 1). Group allocation was determined following blinded review of discharge records by 2 attending neurologists. No propensity score matching was employed, given the homogeneity of primary diagnosis and uniformity in treatment protocols within the unit.

**Figure 2.**
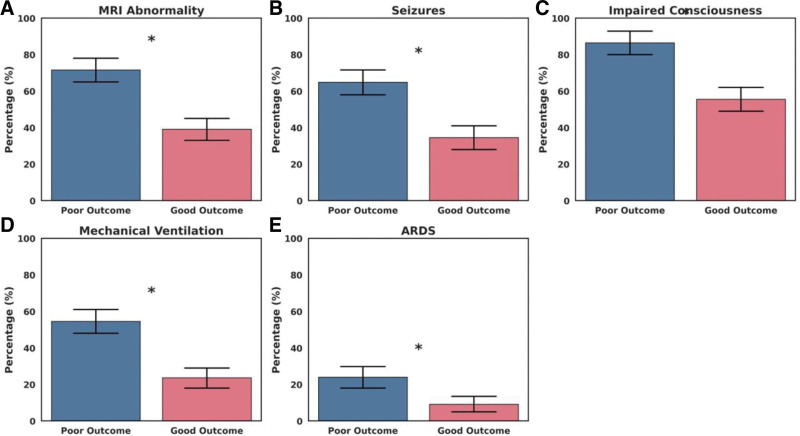
Comparison of significant clinical characteristics between poor and good outcome groups in children with influenza-associated encephalopathy. (A) MRI abnormalities were more prevalent in the poor outcome group. (B) Seizure occurrence was significantly higher among poor prognosis patients. (C) Impaired consciousness was common in the poor outcome group. (D) Requirement for mechanical ventilation was greater in patients with poor outcomes. (E) The incidence of acute ARDS was significantly higher in the poor outcome group. Bars represent the proportion of patients with 95% CIs. “Poor outcome” was defined as death or neurological sequelae at discharge. Asterisks (*) denote statistically significant differences between groups (*P* < .05). ARDS = acute respiratory distress syndrome, CI = confidence interval, MRI = magnetic resonance imaging.

Across the cohort, significant differences emerged in baseline physiological and laboratory parameters (Table 1). Children with poor outcomes presented at younger median age (20 vs 30 months, *P* = .004) and had lower body weight (11.3 ± 3.7 kg vs 13.5 ± 4.2 kg, *P* < .001). Systolic and diastolic blood pressures were modestly reduced in the adverse outcome group (92 and 56 mm Hg, respectively), with both variables reaching statistical significance. Metabolic derangements were more pronounced among poor prognosis cases, reflected in elevated serum glucose (7.1 ± 2.4 mmol/L, *P* = .018) and lower serum sodium (133.4 ± 4.1 mmol/L, *P* = .001). Cardiac biomarkers showed greater perturbation, particularly BNP (366 pg/mL vs 204 pg/mL, *P* = .007) and troponin-T (38 ng/L vs 24 ng/L, *P* = .011). Preexisting comorbidities were more common in this group, notably chronic neurological disorders (12.5% vs 3.6%, *P* = .018). Moreover, compared with the favorable outcome group, children with poor outcome more frequently exhibited low GCS scores, seizures, impaired consciousness, abnormal MRI and electroencephalogram findings, as well as higher rates of ARDS, septic shock, mechanical ventilation, and the use of corticosteroids or IVIG (Table 2). The distribution of these disparities is further illustrated in visual analyses, which depict the proportion of patients meeting critical clinical thresholds across outcome strata (Fig. 2A–E).

**Table 1:** Baseline characteristics of children with influenza-associated encephalopathy according to prognosis.

Variable	Poor outcome (n = 88)	Good outcome (n = 110)	Test (value)	*P* value
Age, mo	20 (10–36)	30 (18–52)	*U* = 3941.5	.004
Male sex, n (%)	53 (60.2%)	63 (57.3%)	χ² = 0.17	.678
Body weight, kg	11.3 ± 3.7	13.5 ± 4.2	*t* = 3.65	<.001
Systolic BP, mm Hg	92 (84–101)	98 (89–106)	*U* = 3756.0	.018
Diastolic BP, mm Hg	56 (49–64)	61 (53–68)	*U* = 3820.0	.023
Blood glucose, mmol/L	7.1 ± 2.4	6.3 ± 1.9	*t* = 2.39	.018
Serum sodium, mmol/L	133.4 ± 4.1	135.6 ± 3.7	*t* = 3.39	.001
ALT, U/L	47 (28–79)	36 (21–58)	*U* = 4085.5	.047
Serum creatinine, μmol/L	42 (35–58)	39 (32–49)	*U* = 4148.0	.065
Troponin-T, ng/L	38 (22–88)	24 (15–42)	*U* = 3620.0	.011
BNP, pg/mL	366 (180–822)	204 (98–412)	*U* = 3502.0	.007
Any comorbidity, n (%)	38 (43.2%)	28 (25.5%)	χ² = 7.06	.008
Neurological disease	11 (12.5%)	4 (3.6%)	χ² = 5.59	.018
Congenital heart disease	9 (10.2%)	7 (6.4%)	χ² = 0.89	.346
Chronic lung disease	7 (8.0%)	5 (4.5%)	χ² = 0.87	.352
Seasonal influenza vaccination	2 (2.3%)	7 (6.4%)	χ² = 1.97	.161

Continuous variables are presented as median (interquartile range) or mean ± standard deviation, based on distribution. Poor outcome was defined as in-hospital death or neurological sequelae at discharge.

ALT = alanine aminotransferase, BNP = brain natriuretic peptide, BP = blood pressure.

**Table 2:** Disease-specific characteristics and management interventions of influenza-associated encephalopathy patients by prognosis.

Variable	Poor outcome (n = 88)	Good outcome (n = 110)	Test (value)	*P* value
Days from fever to admission	2.0 (1.0–3.0)	2.0 (1.0–2.0)	*U* = 4456.0	.164
Initial GCS score	7 (5–10)	12 (10–14)	*U* = 2983.0	<.001
Seizures, n (%)	57 (64.8%)	38 (34.5%)	χ² = 15.36	<.001
Impaired consciousness	76 (86.4%)	61 (55.5%)	χ² = 20.38	<.001
Abnormal brain MRI	63 (71.6%)	43 (39.1%)	χ² = 19.65	<.001
EEG abnormalities	42 (47.7%)	28 (25.5%)	χ² = 9.48	.002
Complicated ARDS	21 (23.9%)	10 (9.1%)	χ² = 8.37	.004
Septic shock	12 (13.6%)	4 (3.6%)	χ² = 5.57	.018
Mechanical ventilation	48 (54.5%)	26 (23.6%)	χ² = 17.98	<.001
Corticosteroid therapy	46 (52.3%)	30 (27.3%)	χ² = 11.40	.001
IVIG therapy	35 (39.8%)	19 (17.3%)	χ² = 11.03	.001

Clinical features were assessed at PICU admission or during hospitalization.

ARDS = acute respiratory distress syndrome, EEG = electroencephalogram, GCS = Glasgow Coma Scale, IVIG = intravenous immunoglobulin, MRI = magnetic resonance imaging, PICU = pediatric intensive care unit.

### 3.2. Prognostic event composition and differential use of critical interventions

**Figure 3.**
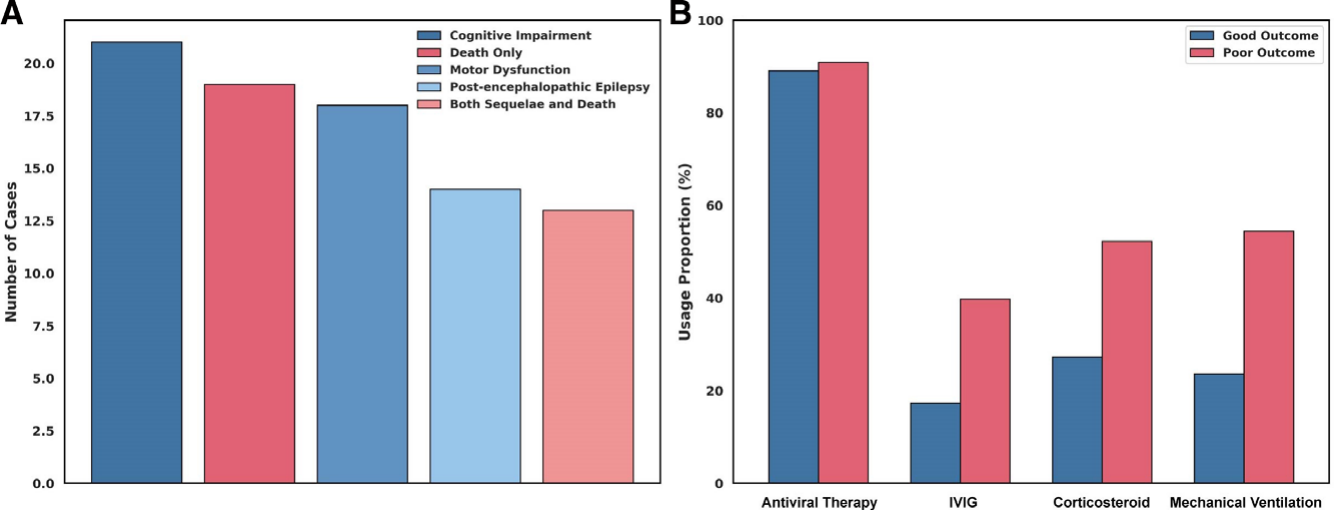
Prognostic event composition and therapeutic interventions among children with poor outcomes. (A) Bar plot displays the frequency of distinct adverse outcomes in the poor prognosis group (n = 88), including cognitive impairment, motor dysfunction, post-encephalopathic epilepsy, death only, and combined neurological sequelae with mortality. (B) Grouped bar chart compares the use of major therapeutic interventions between poor and good outcome groups, including mechanical ventilation, corticosteroids, IVIG, and antiviral agents. Event categories in (A) are not mutually exclusive; some patients experienced multiple adverse outcomes simultaneously. Treatment patterns in (B) reflect differential clinical severity and escalation of care in intensive settings. IVIG = intravenous immunoglobulin.

Among the 88 children classified as having poor outcomes, the final adverse event was heterogeneous in both type and severity (Table 3). Neurological sequelae constituted the predominant contributor, affecting 60.2% of these patients at discharge. The most frequently observed impairments were cognitive dysfunction (23.9%), motor disability (20.5%), and post-encephalopathic epilepsy (15.9%). Notably, mortality was recorded in 21.6% of cases, while an additional 14.8% exhibited both persistent neurological deficits and death following care withdrawal or deterioration. These categories were not mutually exclusive; several patients experienced overlapping end points (Fig. 3A).

**Table 3:** Detailed composition of poor prognosis outcomes.

Event type	Count (n)	Proportion (%)
Cognitive impairment	21	23.9 (21/88)
Motor dysfunction	18	20.5 (18/88)
Post-encephalopathic epilepsy	14	15.9 (14/88)
Death only	19	21.6 (19/88)
Both sequelae and death	13	14.8 (13/88)

Percentages are calculated as proportions of the poor outcome group (n = 88). The categories of sequelae and death are not mutually exclusive; overlapping classification was permitted in cases of co-occurrence.

Therapeutic interventions varied significantly between the prognostic groups and were reflective of the underlying severity profiles (Fig. 3B). In the poor outcome cohort, mechanical ventilation was administered in over half of cases (54.5%), in contrast to less than one-quarter among those with favorable recovery (23.6%). Similarly, corticosteroid pulse therapy and IVIG were more commonly employed in patients with adverse outcomes (52.3% and 39.8%, respectively), likely in response to fulminant disease courses and suspected neuroinflammatory mechanisms. In contrast, antiviral therapy was uniformly applied in both groups, exceeding 89% adherence, consistent with standard protocol for confirmed influenza-associated disease. The divergence in intervention patterns underscores the intensive care burden associated with poor prognostic trajectories in pediatric IAE.

### 3.3. Univariable analysis of predictors of poor prognosis

**Figure 4.**
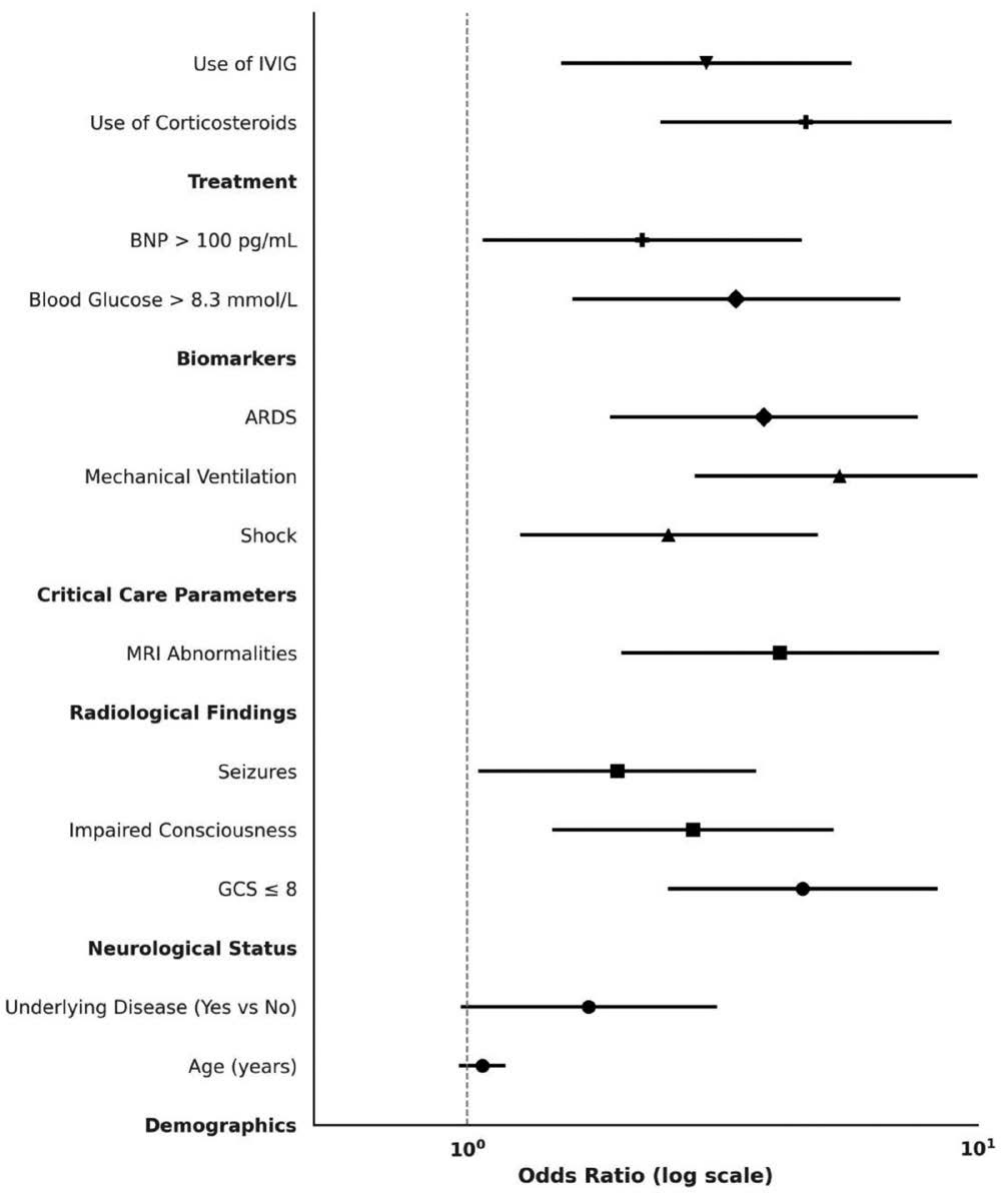
Univariable logistic regression of risk factors for poor prognosis in pediatric influenza-associated encephalopathy patients. Forest plot illustrating the ORs and 95% CIs for selected clinical and laboratory variables associated with poor prognosis, based on univariable logistic regression. Variables are grouped by clinical domains for clarity, including demographics, neurological status, radiological findings, intensive care parameters, biomarkers, and therapeutic interventions. The vertical dashed line indicates the null value (OR = 1.0). Bold labels represent categorical domains; individual risk factors are listed as subordinate rows. CIs are capped by double-ended horizontal bars for visual clarity. ARDS = acute respiratory distress syndrome, BNP = brain natriuretic peptide, CI = confidence interval, GCS = Glasgow Coma Scale, IVIG = intravenous immunoglobulin, MRI = magnetic resonance imaging, OR = odds ratio.

To preliminarily identify risk factors associated with poor outcomes in pediatric patients with IAE, univariable logistic regression was performed across a panel of clinically relevant variables (Table 4, Fig. 4). Among demographic characteristics, neither age nor the presence of underlying comorbidities reached conventional thresholds for statistical significance, although the latter approached marginal association (OR 1.73, 95% CI: 0.98–3.06, *P* = .061). In contrast, neurological status at admission emerged as a strong predictor of adverse prognosis. A GCS score of ≤ 8 was associated with a nearly fivefold increase in risk (OR 4.54, 95% CI: 2.49–8.29, *P* < .001), while altered consciousness and seizures were also significantly linked to poor outcomes (ORs 2.77 and 1.97, respectively).

**Table 4:** Univariable logistic regression analysis of factors associated with poor prognosis.

Variable	β coefficient	OR (95% CI)	*P* value
Age (yr)	0.07	1.07 (0.97–1.18)	.18
Underlying disease (yes vs no)	0.55	1.73 (0.98–3.06)	.061
GCS ≤ 8 (yes vs no)	1.51	4.54 (2.49–8.29)	<.001
Impaired consciousness (yes vs no)	1.02	2.77 (1.48–5.18)	.001
Seizures (yes vs no)	0.68	1.97 (1.06–3.65)	.032
MRI abnormalities (yes vs no)	1.41	4.10 (2.02–8.33)	<.001
Shock (yes vs no)	0.91	2.48 (1.28–4.83)	.007
Mechanical ventilation (yes vs no)	1.68	5.37 (2.81–10.27)	<.001
ARDS (yes vs no)	1.34	3.81 (1.92–7.58)	<.001
Blood glucose > 8.3 mmol/L (yes vs no)	1.21	3.36 (1.62–6.99)	.001
BNP > 100 pg/mL (yes vs no)	0.79	2.20 (1.08–4.49)	.029
Use of corticosteroids (yes vs no)	1.53	4.61 (2.41–8.83)	<.001
Use of IVIG (yes vs no)	1.08	2.94 (1.54–5.62)	.001

ORs and β coefficients were derived from univariable logistic regression models assessing the association between candidate risk factors and poor prognosis, defined as in-hospital death and/or presence of neurological sequelae at discharge. ORs are presented with 95% CIs. Variables were selected based on clinical relevance and significant intergroup differences in prior sections.

ARDS = acute respiratory distress syndrome, BNP = brain natriuretic peptide, CI = confidence interval, GCS = Glasgow Coma Scale, IVIG = intravenous immunoglobulin, MRI = magnetic resonance imaging, OR = odds ratio.

Among radiographic and intensive care parameters, abnormalities on cranial MRI conferred more than a fourfold increase in odds of adverse prognosis (OR 4.10, 95% CI: 2.02–8.33, *P* < .001). The need for mechanical ventilation was one of the strongest predictors identified (OR 5.37, 95% CI: 2.81–10.27, *P* < .001), followed by the presence of ARDS (OR 3.81, *P* < .001) and hemodynamic shock (OR 2.48, *P* = .007). Elevated serum glucose (> 8.3 mmol/L) and BNP (> 100 pg/mL) were each significantly associated with higher odds of poor prognosis (ORs 3.36 and 2.20, respectively). Lastly, both corticosteroid use and IVIG administration demonstrated strong associations with adverse outcomes (ORs 4.61 and 2.94), likely reflecting their application in patients with more severe presentations. These findings informed the selection of variables for subsequent multivariable modeling.

### 3.4. Multivariable model for independent risk factors

**Figure 5.**
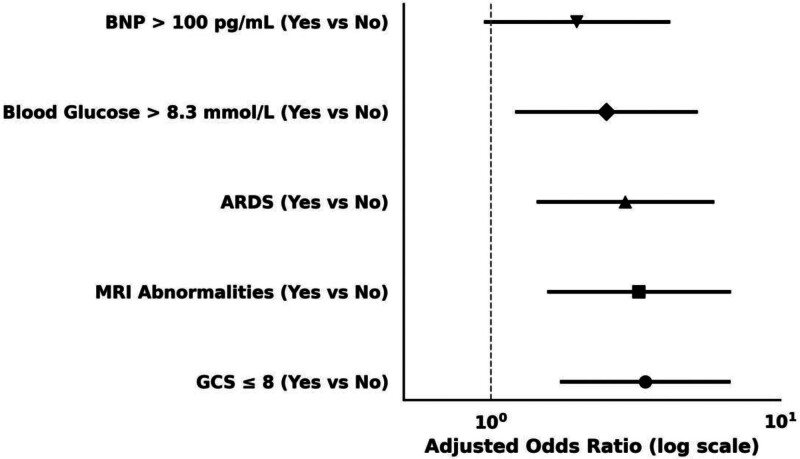
Final multivariable logistic regression model identifying independent predictors of poor prognosis. Forest plot demonstrating aORs and 95% CIs for the 5 independent predictors of poor prognosis in children with influenza-associated encephalopathy, derived from the final multivariable logistic regression model. Variables were selected based on clinical relevance and statistical significance in prior univariable analysis. Each point estimate represents the aOR with a corresponding 95% CI, visually encoded using double-capped horizontal error bars. The vertical dashed line indicates the null value (aOR = 1). The model included GCS ≤ 8, cranial MRI abnormalities, ARDS, elevated blood glucose (> 8.3 mmol/L), and BNP (> 100 pg/mL). aOR = adjusted odds ratio, ARDS = acute respiratory distress syndrome, BNP = brain natriuretic peptide, CI = confidence interval, GCS = Glasgow Coma Scale, MRI = magnetic resonance imaging.

To isolate prognostic determinants independent of potential confounding, multivariable logistic regression was conducted incorporating 5 variables identified through univariable analysis and established clinical relevance (Table 5, Fig. 5). After covariate adjustment, impaired neurological status at admission remained one of the most robust predictors of poor prognosis. Patients presenting with a GCS score ≤ 8 demonstrated a 3.42-fold increased likelihood of adverse outcomes compared to those with higher scores (aOR 3.42, 95% CI: 1.76–6.64, *P* < .001). Similarly, the presence of abnormal cranial MRI findings retained a significant association with poor prognosis (aOR 3.25, 95% CI: 1.59–6.67, *P* = .001).

**Table 5:** Multivariable logistic regression of predictors of poor prognosis.

Variable	β coefficient	Adjusted OR (95% CI)	*P* value
GCS ≤ 8 (yes vs no)	1.23	3.42 (1.76–6.64)	<.001
MRI abnormalities (yes vs no)	1.18	3.25 (1.59–6.67)	.001
ARDS (yes vs no)	1.07	2.92 (1.46–5.85)	.002
Blood glucose > 8.3 mmol/L (yes vs no)	0.92	2.51 (1.23–5.12)	.011
BNP > 100 pg/mL (yes vs no)	0.68	1.98 (0.96–4.12)	.065

ORs and β coefficients were derived from univariable logistic regression models assessing the association between candidate risk factors and poor prognosis, defined as in-hospital death and/or presence of neurological sequelae at discharge. ORs are presented with 95% CIs. This multivariable logistic regression model included 5 covariates selected for their clinical significance and univariable relevance. The strongest independent predictors of poor prognosis were GCS ≤ 8, cranial MRI abnormalities, and ARDS, each demonstrating adjusted odds ratios above 2.9 with high statistical significance. Elevated serum glucose was also independently associated with poor outcomes. BNP > 100 pg/mL showed a borderline association (*P* = .065) and was retained for model completeness given its physiologic relevance. These variables form the basis for predictive modeling in subsequent analysis.

ARDS = acute respiratory distress syndrome, BNP = brain natriuretic peptide, CI = confidence interval, GCS = Glasgow Coma Scale, MRI = magnetic resonance imaging, OR = odds ratio.

Among intensive care indicators, the development of ARDS was independently associated with increased risk (aOR 2.92, 95% CI: 1.46–5.85, *P* = .002), supporting its value as a marker of systemic severity. Biochemical parameters including hyperglycemia (serum glucose > 8.3 mmol/L) also contributed meaningfully to the model, conferring a more than twofold elevated risk (aOR 2.51, 95% CI: 1.23–5.12, *P* = .011). Although elevated BNP (> 100 pg/mL) demonstrated only a borderline association (aOR 1.98, 95% CI: 0.96–4.12, *P* = .065), it was retained in the final model given its physiological plausibility and potential predictive contribution. These 5 variables were subsequently incorporated into a composite prognostic framework.

### 3.5. Development and performance of the prognostic prediction model

**Figure 6.**
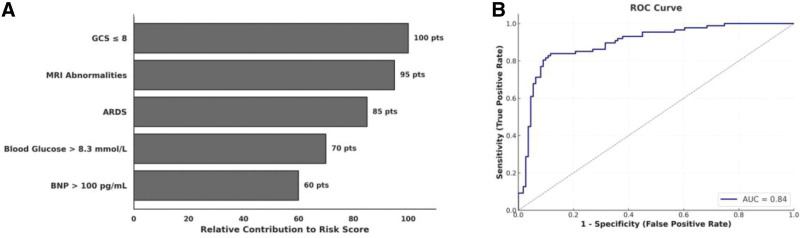
Visualized prognostic model for predicting poor outcomes in pediatric IAE. (A) Nomogram integrating 5 independent predictors derived from multivariable logistic regression, each weighted by its relative contribution to the composite risk score. Total scores correspond to estimated individual probabilities of poor prognosis. (B) ROC curve for the final prediction model. The AUC was 0.84, indicating strong discriminative capacity. The nomogram in (A) was constructed using 5 independent risk factors: GCS ≤ 8, cranial MRI abnormalities, ARDS, hyperglycemia (> 8.3 mmol/L), and elevated BNP (> 100 pg/mL). Point allocations reflect each variable’s proportional contribution to risk estimation. The ROC analysis in (B) was based on 198 patients, including 88 with poor prognostic events, and demonstrated a high predictive accuracy of the model. ARDS = acute respiratory distress syndrome, AUC = area under the curve, BNP = brain natriuretic peptide, GCS = Glasgow Coma Scale, IAE = influenza-associated encephalopathy, MRI = magnetic resonance imaging, ROC = receiver operating characteristic.

A prognostic model was constructed incorporating the 5 independently associated variables from the multivariable logistic regression analysis: GCS ≤ 8, abnormal cranial MRI findings, ARDS, blood glucose > 8.3 mmol/L, and BNP > 100 pg/mL. Each predictor was assigned a weighted point value based on its β coefficient, enabling generation of a composite risk score for each patient (Fig. 6A). The nomogram visualization reflects the relative contribution of each factor, with neurological severity and radiographic abnormalities contributing the greatest weight.

Model discrimination was assessed via ROC curve analysis. The final model demonstrated a high degree of discriminative power for identifying children at risk of poor prognosis, with an area under the curve of 0.84, a sensitivity of 82.3%, and a specificity of 88.2% at the optimal cutoff (Fig. 6B). This indicates strong sensitivity and specificity across a range of threshold probabilities. The ROC curve was derived from the entire cohort of 198 patients, of whom 88 experienced adverse outcomes as previously defined. Internal validation metrics support the robustness of this model for risk stratification in critically ill pediatric patients with IAE.

## 4. Discussion

In this retrospective cohort study of 198 children with IAE admitted to a PICU over an 8-year period, we found that nearly half of the patients experienced a poor in-hospital outcome, defined as death, persistent coma, or newly developed neurological sequelae. Among these, cognitive deficits, motor dysfunction, and post-encephalopathic epilepsy were the most prevalent. Five routinely accessible clinical variables – reduced GCS score, cranial MRI abnormalities, ARDS, hyperglycemia, and elevated BNP levels – were independently associated with adverse outcomes. A multivariable prognostic model incorporating these variables demonstrated strong discriminatory performance, and was translated into a nomogram with potential clinical utility for early risk stratification.

These findings align partially with prior reports that have highlighted the prognostic significance of neurological status and radiological abnormalities in IAE.^[1,2,15]^ Reduced GCS has consistently been associated with poor outcomes in both pediatric and adult encephalopathies.^[16,17]^ Similarly, neuroimaging abnormalities – particularly bilateral thalamic involvement and cortical lesions – are recognized as indicators of severe disease, though the heterogeneity of radiological patterns in IAE has limited their predictive specificity.^[14,18]^ Our study contributes to this body of evidence by confirming the prognostic value of these indicators within a larger and more systematically defined cohort. Moreover, we observed associations between poor prognosis and systemic complications, notably ARDS and metabolic disturbances. Hyperglycemia and elevated BNP, while not specific to neurological injury,^[19,20]^ may reflect broader physiologic stress or multiorgan involvement, supporting their inclusion in risk models aimed at critically ill pediatric populations.

The nomogram derived from this analysis integrates variables commonly available within the first 24 hours of PICU admission, and may assist clinicians in estimating short-term risk with minimal delay. While not intended to substitute clinical judgment, such tools can aid decision-making around intensive monitoring, parental counseling, and resource allocation. Unlike previous studies that have focused primarily on mortality, our endpoint encompasses a broader range of disabling outcomes, thereby offering a more nuanced perspective on recovery trajectories. The incorporation of discharge neurological status acknowledges the substantial burden posed by cognitive and motor sequelae, which may persist well beyond the acute phase.

This study has several limitations. Its retrospective and single-center nature may introduce selection bias and limit generalizability. Additionally, the lack of long-term follow-up precludes definitive conclusions regarding the durability of neurological recovery or progression. Furthermore, as a single-center observational study, although we identified risk factors associated with poor outcomes, rigorously designed multicenter case-control studies or prospective cohort studies are necessary to further validate these factors – particularly the potential protective effect of seasonal influenza vaccination against severe neurological complications. Data on viral subtypes, inflammatory biomarkers, and detailed neurocognitive assessments were unavailable, constraining mechanistic insights. Future studies should focus on conducting more detailed subgroup analyses based on influenza virus serotypes (influenza A subtypes vs B) and vaccination status in larger cohorts, particularly regarding clinical manifestations such as febrile seizure subtypes. This would help clarify the associations between specific viral strains or immune backgrounds and distinct neurological phenotypes. External validation of the model in independent cohorts will be essential before routine implementation.

Nevertheless, this study represents one of the largest efforts to date to characterize the clinical trajectory of pediatric IAE in a high-acuity setting, and to construct a pragmatic tool for prognostic stratification. Future research should focus on multicenter validation, prospective data collection, and the integration of biological markers to refine predictive precision and enhance the personalization of care.

## Author contributions

**Conceptualization:** Hong Cheng, Hong Zhang.

**Data curation:** Hui Li.

**Formal analysis:** Hui Li.

**Investigation:** Hui Li.

**Methodology:** Hong Zhang.

**Writing – original draft:** Fei Xiao, Xingfeng Cheng.

**Writing – review & editing:** Yuanmei Shi, Kang Xu.

## Supplementary Material



## References

[1] Song Y Li S Xiao W. Influenza-associated encephalopathy and acute necrotizing encephalopathy in children: a retrospective single-center study. Med Sci Monitor. 2021;27:e928374–928371. 10.12659/MSM.928374 PMC7789050 33388740

[2] Yang M Yi L Jia F Zeng X Liu Z. Characteristics and outcome of influenza-associated encephalopathy/encephalitis among children in China. Clinics (Sao Paulo, Brazil). 2024;79:100475. 39096859 10.1016/j.clinsp.2024.100475 PMC11345302

[3] Bi J Wu X Deng J. Mortality risk factors in children with influenza‐associated encephalopathy admitted to the paediatric intensive care unit between 2009 and 2021. J Paediatr Child Health. 2024;60:456–61. 39022988 10.1111/jpc.16611

[4] Cleuziou P Renaldo F Renolleau S. Mortality and neurologic sequelae in influenza-associated encephalopathy: retrospective multicenter PICU cohort in France. Pediatr Critical Care Med. 2021;22:e582–7. 33950890 10.1097/PCC.0000000000002750

[5] Kimura-Ohba S Kitamura M Tsukamoto Y. Viral entry and translation in brain endothelia provoke influenza-associated encephalopathy. Acta Neuropathol. 2024;147:77. 38687393 10.1007/s00401-024-02723-z PMC11061015

[6] Donaldson AL Hardstaff JL Harris JP Vivancos R O’brien S. School-based surveillance of acute infectious disease in children: a systematic review. BMC Infect Dis. 2021;21:744. 34344304 10.1186/s12879-021-06444-6 PMC8330200

[7] Donnelley E Teutsch S Zurynski Y. Severe influenza-associated neurological disease in Australian children: seasonal population-based surveillance 2008–2018. J Pediatric Infect Dis Soc. 2022;11:533–40. 36153667 10.1093/jpids/piac069

[8] Auerbach SR Richmond ME Schumacher KR. Infectious complications of ventricular assist device use in children in the United States: data from the pediatric interagency registry for mechanical circulatory support (pedimacs). J Heart Lung Transpl. 2018;37:46–53. 10.1016/j.healun.2017.09.013 PMC5849428 29107545

[9] Dumaine C Bekkar S Belot A. Infectious adverse events in children with juvenile idiopathic arthritis treated with biological agents in a real-life setting: data from the JIRcohorte. Joint Bone Spine. 2020;87:49–55. 31369865 10.1016/j.jbspin.2019.07.011

[10] Sonneville R Jaquet P Vellieux G de Montmollin E Visseaux B. Intensive care management of patients with viral encephalitis. Rev Neurol (Paris). 2022;178:48–56. 34973832 10.1016/j.neurol.2021.12.002

[11] Verity C Baker E Maunder P Pal S Winstone AM. Differential diagnosis of progressive intellectual and neurological deterioration in children. Develop Med Child Neurol. 2021;63:287–94. 32970345 10.1111/dmcn.14691 PMC7891454

[12] Riviello JJ Jr Ashwal S Hirtz D. Practice parameter: diagnostic assessment of the child with status epilepticus (an evidence-based review) report of the quality standards subcommittee of the American academy of neurology and the practice committee of the child neurology society. Neurology. 2006;67:1542–50. 17101884 10.1212/01.wnl.0000243197.05519.3d

[13] Knight S Takagi M Fisher E. A systematic critical appraisal of evidence-based clinical practice guidelines for the rehabilitation of children with moderate or severe acquired brain injury. Arch Phys Med Rehabil. 2019;100:711–23. 29966649 10.1016/j.apmr.2018.05.031

[14] Abend NS Licht DJ. Predicting outcome in children with hypoxic ischemic encephalopathy. Pediatr Crit Care Med. 2007;8:1–8. 18477911 10.1097/01.PCC.0000288714.61037.56

[15] Mizuguchi M Yamanouchi H Ichiyama T Shiomi M. Acute encephalopathy associated with influenza and other viral infections. Acta Neurol Scand. 2007;115:45–56. 17362276 10.1111/j.1600-0404.2007.00809.x

[16] Balakrishnan B VanDongen-Trimmer H Kim I. GCS-pupil score has a stronger association with mortality and poor functional outcome than GCS alone in pediatric severe traumatic brain injury. Pediatr Neurosurg. 2021;56:432–9. 34284393 10.1159/000517330

[17] Emami P Czorlich P Fritzsche FS. Impact of Glasgow Coma Scale score and pupil parameters on mortality rate and outcome in pediatric and adult severe traumatic brain injury: a retrospective, multicenter cohort study. J Neurosurg. 2017;126:760–7. 27035177 10.3171/2016.1.JNS152385

[18] Imataka G Kuwashima S Yoshihara S. A comprehensive review of pediatric acute encephalopathy. J Clin Med. 2022;11:5921. 36233788 10.3390/jcm11195921 PMC9570744

[19] Dillinger J-G Patin C Bonnin P. Elevated brain natriuretic peptide and high brachial pulse pressure in patients with diabetes. Am J Hypertens. 2022;35:414–22. 34969077 10.1093/ajh/hpab179

[20] Chang P Zhang X Zhang J. BNP protects against diabetic cardiomyopathy by promoting Opa1-mediated mitochondrial fusion via activating the PKG-STAT3 pathway. Redox Biol. 2023;62:102702. 37116257 10.1016/j.redox.2023.102702 PMC10165144

